# Surveillance for *Ixodes scapularis* and *Ixodes pacificus* ticks and their associated pathogens in Canada, 2021

**DOI:** 10.14745/ccdr.v52i0102a03

**Published:** 2026-02-19

**Authors:** Safa Ahmad, Gamal Wafy, Christy Wilson, Heather Coatsworth, Camille Guillot, Jade Savage, Patrick Leighton, Priya Goundar, Muhammad Morshed, Peter Buck, Annie-Claude Bourgeois, Salima Gasmi

**Affiliations:** 1 Centre for Food-borne, Environmental and Zoonotic Infectious Diseases, Public Health Agency of Canada, Ottawa, ON; 2 National Microbiology Laboratory Branch, Public Health Agency of Canada, Winnipeg, MB; 3 Canadian Lyme Disease Research Network, University of Guelph, Guelph, ON; 4 eTick, Bishop’s University, Sherbrooke, QC; 5 Ministry of Health, Regina, SK; 6 Public Health Laboratory, BC Centre for Disease Control, Vancouver, BC; 7 Department of Pathology and Laboratory Medicine, University of British Columbia, Vancouver, BC; 8 Centre for Food-borne, Environmental and Zoonotic Infectious Diseases, Public Health Agency of Canada, Saint-Hyacinthe, QC

**Keywords:** *Ixodes scapularis*, *Ixodes pacificus*, surveillance, Borrelia, Anaplasma, Babesia, Powassan virus

## Abstract

**Background:**

*Ixodes scapularis* and *Ixodes pacificus* ticks pose risk of infection with tick-borne diseases in Eastern and Pacific Western Canada, respectively.

**Objective:**

In 2021, passive and active tick surveillance programs collected ticks and associated data elements, including location, infection and other characteristics, to monitor their populations and inform public health prevention and mitigation activities.

**Methods:**

Surveillance data for ticks were compiled from the National Microbiology Laboratory (Public Health Agency of Canada), provincial public health, Canadian Lyme Disease Research Network and eTick (an image-based online platform). A descriptive analysis of tick records and infection prevalence of tick-borne pathogens is presented. Seasonal trends are described.

**Results:**

During 2021, 6,892 *I. scapularis* ticks were identified across all ten provinces via passive surveillance with 777 *I. pacificus* ticks collected from British Columbia. Most were adult female ticks, collected from human hosts in the spring (March–May) or fall (October–November) seasons. The most common pathogen, *Borrelia burgdorferi,* was detected in 18.6% (95% CI: 17.2%–20.1%) of samples. Active surveillance resulted in 1,929 *I. scapularis* and 18 *I. pacificus* ticks collected in six provinces. Among *I. scapularis*, 22.3% were infected with *B. burgdorferi*, 11.8% with *Babesia odocoilei* and 4.3% with *Anaplasma phagocytophilum*. Fewer than 1% were infected with each of *Borrelia miyamotoi* (0.7%), *Babesia microti* (0.1%) and Powassan virus (0.1%).

**Conclusion:**

As the risk of infection with tick-borne diseases continues to grow in many parts of Canada, monitoring trends in infection prevalence and the geographical range expansion of ticks provides essential data to inform public health actions and messaging.

## Introduction

*Ixodes scapularis* and *Ixodes pacificus* ticks are known to transmit several bacterial, viral and protozoan pathogens to humans in Eastern/Central and Western Canada, respectively. They are doing so in increasing population numbers and across a broader geographical range due to climate and environmental changes ((1–5)). The resulting increase in potential for tick-borne diseases in the country, especially in Southern Central and Eastern Canada, has been emphasized in previous reports and in research literature and requires ongoing surveillance so prevention efforts can be successful ((1,6–8)). Case numbers of Lyme disease reported in Canada in 2022 have increased more than sevenfold since 2012 ((9)). Additional tick-borne diseases transmitted by *I. scapularis* or *I. pacificus*, namely, anaplasmosis, babesiosis and Powassan virus disease, are nationally notifiable diseases in Canada as of early 2024 ((10–13)).

Although tick surveillance has been conducted in Canada since the 1990s, data started to be summarized annually at the national-level by the Centre for Food-borne, Environmental and Zoonotic Infectious Diseases, Public Health Agency of Canada (PHAC) in 2019, and provide a baseline for tick-borne disease risk that, over time, will help to identify trends ((14)).

The objective of this annual surveillance report is to update the summary of characteristics of the main Lyme disease vectors in Canada, *I. scapularis* and *I. pacificus*, collected through passive and active surveillance during 2021. This article also summarizes the prevalence and spatial distribution of their associated pathogens.

## Methods

### Data sources

This report uses two types of surveillance data from more than 20 different providers. Passive surveillance datasets were provided by the National Microbiology Laboratory (NML) branch of PHAC, British Columbia Centre for Disease Control, Saskatchewan Ministry of Health and eTick. Active surveillance datasets were provided by the Canadian Lyme Disease Research Network, 12 Ontario health units, *Laboratoire de santé publique du Québec*, University of Manitoba, Manitoba Health, Seniors and Long-Term Care Department, New Brunswick Department of Health, University of New Brunswick and University of Ottawa.

**Passive tick surveillance:** As in 2020, this analysis was limited to *I. scapularis* and *I. pacificus* collected in Canada in the pertinent year ((6)). Provinces with five or fewer ticks of a given species submitted for species identification and laboratory testing were excluded to avoid misinterpretation of results. Ticks with a location of acquisition outside of the province of submission were not geocoded.

Additional regional passive tick surveillance programs have been discontinued since the publication of the previous report due to laboratory capacity constraints and as *I. scapularis* populations have become established. As before, ticks (or their images) acquired in these jurisdictions could be submitted by the public directly to NML or eTick.

eTick is a web-based, community-science project inviting the public to help with population tick monitoring and is used as a passive surveillance system for ticks in Canada ((15)). Individuals submit images of ticks they encounter online or via the mobile application, which are then examined by trained personnel to identify the species. Only one tick can be submitted in a single image-based submission.

Ticks collected and submitted from Alberta, Manitoba, Ontario, Québec, New Brunswick and Nova Scotia and tested for *Anaplasma phagocytophilum*, *Borrelia burgdorferi*, *Borrelia miyamotoi* and *Babesia microti* at the NML using methods previously described were included in this report ((16,17)). Among ticks tested by the British Columbia Centre for Disease Control, only results for *B. burgdorferi* were included in this report. Additional details regarding methodology are available in the previously published annual report ((6)).

**Active tick surveillance:** In active surveillance, ticks are collected from the environment using drag sampling or capturing host mammals that are then examined for ticks. This analysis used data from efforts to collect ticks from 10 sites in British Columbia, six in Alberta, at least eight in Saskatchewan, nine sites in Manitoba, more than 60 in Ontario, 36 in Québec, 14 sites in New Brunswick and 10 in Nova Scotia. Drag sampling took place in late spring/summer (May–July) and fall (September–November). Ticks were tested for some or all of the following pathogens: *A. phagocytophilum*; *B. microti*; *B. odocoilei*; *B. burgdorferi*; *B. miyamotoi* and Powassan virus.

### Analysis

**Tick characteristics:** For passive surveillance, descriptive statistics were calculated for submission type (sample-based or image-based), tick species, province of acquisition, stage (larva, nymph, adult female or adult male), level of engorgement (unfed or engorged), host (human, dog, cat or other) and month of collection. For active surveillance, descriptive statistics were calculated for province of collection and stage (larva, nymph, adult female or adult male). All data were cleaned and analysed in R (version 4.0.2).

Ticks submitted through passive surveillance that were acquired in Canada and not associated with a travel history to other provinces or countries were mapped using QGIS software (version 3.34.7) based on their location of acquisition. Ticks submitted with a record of history of travel in the previous 14 days within the same province as the locality of acquisition were geocoded to the submitter-provided location of exposure during travel. In active surveillance, the site location of tick dragging was geocoded from data obtained from the NML and mapped for all data.

**Infection prevalence:** To account for pooled testing of ticks collected by passive surveillance from some jurisdictions, maximum likelihood estimates of prevalence were calculated with 95% confidence intervals (CI) using the PooledInfRate R package (version 1.6) ((18,19)). This estimates the probability of infection for an individual tick in the population using the results of testing of the pooled samples (i.e., a group of one or more ticks submitted and tested together). The package was developed by the United States’ Centers for Disease Control and Prevention ((19)). Co-infection prevalence was calculated among single submissions only to ascertain true co-infections, that is, two or more pathogens in a single tick. Where ticks were not tested in pools, prevalence was the number of positive ticks divided by the number of ticks tested.

## Results

### Passive surveillance tick characteristics

**Figure 1:**
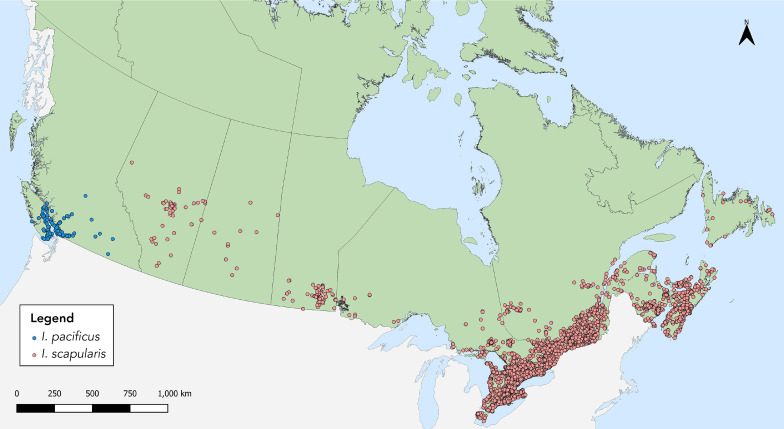
*Ixodes pacificus* and *Ixodes scapularis* ticks submitted through passive tick surveillance, Canada, 2021^a^ ^a^ Each dot represents a probable location of acquisition for a single or multiple submission of *I. scapularis* (n=6,832 submissions) or *I. pacificus* (n=755 submissions) made via passive surveillance programs

In 2021, 7,669 *I. scapularis* (n=6,892) and *I. pacificus* (n=777) ticks were submitted by provinces across Canada, with at least 14 submissions per province (**Table 1**, **Figure 1**). Image-based submissions comprised 54.9% of ticks submitted (n=4,210) and the remainder were sample-based submissions (n=3,459). Ticks from Ontario, Québec and Nova Scotia comprised 83.5% of all ticks submitted. The majority (98.3%) of ticks were from single submissions.

**Table 1:** Number of *Ixodes pacificus* and *Ixodes scapularis* ticks and submissions collected through passive surveillance by province, Canada, 2021^a^

Province	Tick species (number of ticks)			Type of surveillance (number of ticks)		Type of submission^b^ (number of submissions)	
	*Ixodes pacificus*	*Ixodes scapularis*	Total	Sample-based	Image-based^c^	Single submissions	Multiple submissions
British Columbia^d^	777	2	779	696	83	743	12
Alberta	0	78	78	16	62	63	1
Saskatchewan	0	15	15	9	6	13	1
Manitoba^d^	0	90	90	5	85	90	0
Ontario^e^	0	4,415	4,415	1,973	2,442	4,365	23
Québec^e^	0	1,377	1,377	659	718	1,356	9
Newfoundland and Labrador	0	18	18	0	18	18	0
New Brunswick	0	214	214	69	145	214	0
Nova Scotia^e^	0	610	610	32	578	602	4
Prince Edward Island	0	73	73	0	73	73	0
Total	777	6,892	7,669	3,459	4,210	7,537	50

^a^ No ticks were reported in Yukon, Northwest Territories or Nunavut for *I. scapularis* or *I. pacificus*

^b^ Single submissions consist of one tick; multiple submissions consist of two or more ticks submitted together by the same individual

^c^ Where ticks were submitted as both samples and images, image submission records were removed

^d^ Sample-based submissions from Manitoba and *I. scapularis* submissions from British Columbia were excluded from subsequent analyses below since ≤5 ticks were submitted of each

^e^ Passive tick surveillance has been discontinued in some regions of Ontario and Québec and all of Nova Scotia; however, individuals could submit ticks directly to the National Microbiology Laboratory or through eTick

Tick stage, level of engorgement and host were available for 98.2%, 89.3% and 100% of *I. pacificus* records and for 80.3%, 40.0% and 99.9% of *I. scapularis* records, respectively (data not shown in table). Ticks submitted only via eTick did not include information about engorgement. The majority of ticks submitted in sample-based submissions were adult female ticks (*I. pacificus*: 96.2%; *I. scapularis*: 86.8%) (**Table 2**).

**Table 2:** Stage, level of engorgement and host of *Ixodes pacificus* and *Ixodes scapularis* ticks submitted through passive surveillance, Canada, 2021^a,b^

Characteristics	Tick species			
	*Ixodes pacificus*		*Ixodes scapularis*	
	n	%	n	%
**Stage**				
Larva	0	0	24	0.46
Nymph	16	2.09	284	5.48
Adult female	735	96.20	4,501	86.84
Adult male	13	1.70	374	7.22
Total	764	100	5,183	100
**Level of engorgement**				
Engorged	77	11.10	1,144	41.80
Unfed	617	88.90	1,593	58.20
Total	694	100	2,737	100
**Host**				
Human	711	91.50	4,809	69.81
Dog	49	6.31	1,670	24.24
Cat	1	0.13	271	3.93
Other^c^	16	2.06	139	2.02
Total	777	100	6,889	100

^a^ Data are presented for all ticks where available, regardless of whether the tick was part of a single or a multiple submission

^b^ No ticks were reported from the Yukon, Northwest Territories or Nunavut for *I. scapularis* or *I. pacificus*. Passive tick surveillance has been discontinued in the entire province of Nova Scotia and some regions of Ontario and Québec; however, individuals could submit ticks directly to the National Microbiology Laboratory or through eTick from these jurisdictions

^c^ Includes environment, horse, pony, chicken and deer

A larger proportion of *I. scapularis* were engorged upon submission than *I. pacificus* (41.8% vs 11.1%, respectively) (Table 2). Most *I. pacificus* submissions were obtained from human hosts (91.4%) while a majority of *I. scapularis* were obtained from human and dog hosts (69.8% and 24.2%, respectively).

**Figure 2:**
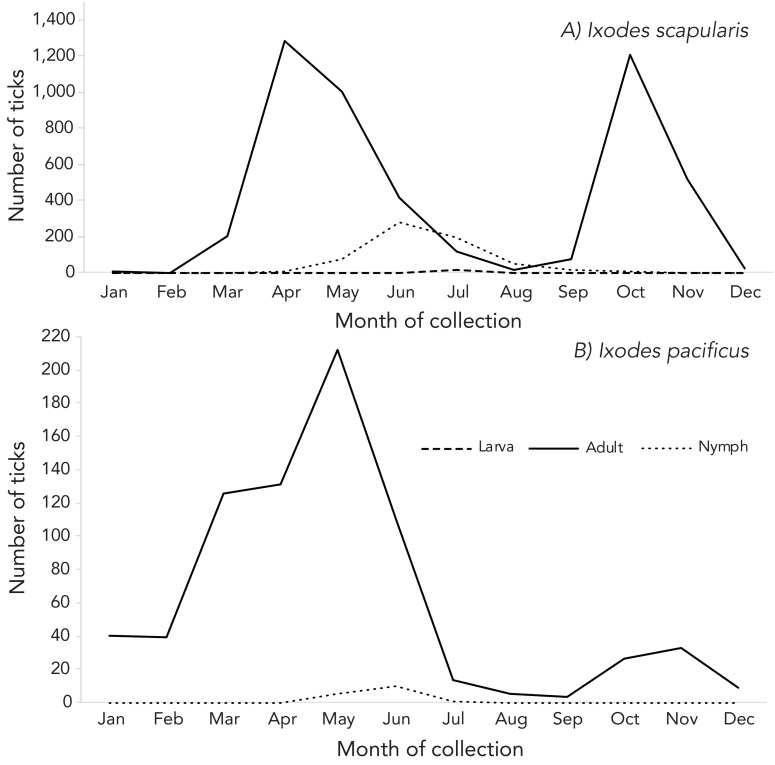
Number of *Ixodes pacificus* and *Ixodes scapularis* ticks submitted through passive surveillance, by month and tick stage, Canada, 2021^a,b^ ^a^ Data are presented for *I. pacificus* (n=764) and *I. scapularis* (n=5,529) ticks submitted through passive surveillance ^b^ No ticks were reported from the Yukon, Northwest Territories or Nunavut for *I. scapularis* or *I. pacificus.* Passive tick surveillance has been discontinued in the entire province of Nova Scotia and some regions of Ontario and Québec; however, individuals could submit ticks directly to the National Microbiology Laboratory or through eTick from these jurisdictions

Records including both month of acquisition and tick stage made up 98.2% of *I. pacificus* and 80.2% of *I. scapularis* submissions (**Figure 2**). All submissions missing tick stage information were image-based submissions, comprising 32.6% of those submissions (n=1,374/4,210).

Adult *I. scapularis* submissions, comprising 80.2% of total *I. scapularis* submitted through passive surveillance, peaked in April and again in October and nymph submissions, comprising 11.4%, peaked in June. For adult *I. pacificus*, submissions peaked in May with a subsequent marginal peak in November.

### Infection prevalence in passive surveillance

Data on laboratory testing was available for 98.6% of *I. pacificus* and 99.4%–99.8% of *I. scapularis* from sample-based submissions, depending on pathogen. Nearly one in five *I. scapularis* ticks in Canada is estimated to be infected with at least one tick-borne pathogen (*A. phagocytophilum*, *B. burgdorferi*, *B. miyamotoi* or *B. microti*) (95% CI: 18.3%–21.3%). The most prevalent pathogen was *B. burgdorferi*, detected in 18.6% of *I. scapularis* (95% CI: 17.2%–20.1%). *Anaplasma phagocytophilum* was detected in 1.1% of *I. scapularis* (95% CI: 0.8%–1.6%). Other tick-borne pathogens were estimated to have a prevalence of fewer than 1% each (0.04% positive for *B. microti* (95% CI: 0.00%–0.18%) and 0.4% positive for *B. miyamotoi* (95% CI: 0.2%–0.7%) (**Table 3**).

**Table 3:** Prevalence of *Anaplasma phagocytophilum*, *Babesia microti*, *Borrelia burgdorferi* and *Borrelia miyamotoi* infection in *Ixodes scapularis* ticks submitted through passive surveillance, Canada, 2021^a^

Pathogen	Infection prevalence		
**Single agent**	**Maximum likelihood estimate**		
	**%**		**95% CI**
*A. phagocytophilum*	1.13		0.78–1.57
*B. microti*	0.04		0.00–0.18
*B. burgdorferi*	18.60		17.18–20.09
*B. miyamotoi*	0.36		0.19–0.65
Total single agent	19.79		18.33–21.31
**Co-infection**	**Co-infection rate**		
	**%**	**Number co-infected ticks^b^/number ticks tested**	
*A. phagocytophilum* + *B. microti*	0	0/2,655	
*A. phagocytophilum* + *B. burgdorferi*	0.30	8/2,664	
*A. phagocytophilum* + *B. miyamotoi*	0.04	1/2,655	
*B. microti* + *B. burgdorferi*	0	0/2,655	
*B. microti* + *B. miyamotoi*	0	0/2,655	
*B. burgdorferi* + *B. miyamotoi*	0.08	3/2,655	
Total co-infected^c^	0.38	10/2,664	

Abbreviation: CI, confidence interval

^a^ Number of *I. scapularis* ticks tested for infection prevalence: *A. phagocytophilum* (n=2,752), *B. microti* (n=2,743), *B. burgdorferi* (n=2,752), *B. miyamotoi* (n=2,743). Only ticks submitted in single submissions could be tested for co-infection (n=2,664). Of the ticks that are excluded, 99.7% (4,210/4,221) were image-based submissions to eTick and 0.4% (n=16) were sample-based submissions missing test results

^b^ Numbers are not mutually exclusive due to the presence of a triple co-infection

^c^ This figure includes nine double co-infections [*A. phagocytophilum* + *B. burgdorferi* (n=7), *B. burgdorferi* + *B. miyamotoi* (n=2)] and one triple co-infection

Among 684 *I. pacificus* ticks, 0.9% indicated presence of *B. burgdorferi* (95% CI: 0.4%–1.8%) (**Table 4**). Co-infections in *I. scapularis* ticks were also estimated to have a prevalence of fewer than 1.0% each (Table 3).

**Table 4:** Prevalence of *Anaplasma phagocytophilum*, *Babesia microti*, *Borrelia burgdorferi* and *Borrelia miyamotoi* infection in *Ixodes scapularis* and *Ixodes pacificus* ticks submitted through passive surveillance, by province, Canada, 2021^a,b^

Province	Infection prevalence Maximum likelihood estimate							
	*A. phagocytophilum*		*B. microti*		*B. burgdorferi*		*B. miyamotoi*	
	%	95% CI	%	95% CI	%	95% CI	%	95% CI
***Ixodes pacificus***								
British Columbia	N/A	N/A	N/A	N/A	0.73	0.31–1.70	N/A	N/A
***Ixodes scapularis***								
Alberta	0	0–19.36	0	0–19.36	0	0–19.36	0	0–19.36
Saskatchewan	22.22	6.32–54.74	N/A	N/A	0.00	0.00–29.91	N/A	N/A
Ontario	0.86	0.52–1.35	0	0–0.19	19.20	17.50–20.99	0.30	0.12–0.63
Québec	1.67	0.88–2.88	0.15	0.01–0.73	17.72	14.94–20.80	0.30	0.05–0.99
New Brunswick	1.45	0.26–7.76	0	0–5.27	11.59	5.99–21.25	1.45	0.26–7.76
Nova Scotia	6.23	1.14–18.77	0	0–10.72	28.55	15.14–45.71	3.16	0.18–14.45
Total	1.13	0.78–1.57	0.04	0–0.18	18.60	17.18–20.09	0.36	0.19–0.65

Abbreviations: CI, confidence interval; N/A, not available

^a^ No ticks were reported in Yukon, Northwest Territories or Nunavut for *I. scapularis* or *I. pacificus*. Passive tick surveillance has been discontinued in the entire province of Nova Scotia and some regions of Ontario and Québec; however, individuals could submit ticks directly to the National Microbiology Laboratory or through eTick from these jurisdictions

^b^ Number of ticks tested: British Columbia (n=684), Alberta (n=16), Saskatchewan (n=9), Ontario (n=1,972), Québec (n=664), New Brunswick (n=69), Nova Scotia (n=32). Sample-based submissions from Manitoba and *I. scapularis* tick submissions from British Columbia were excluded due to small sample size (n=fewer than five for each). Only image-based submissions were received from Newfoundland and Labrador and Prince Edward Island

**Figure 3:**
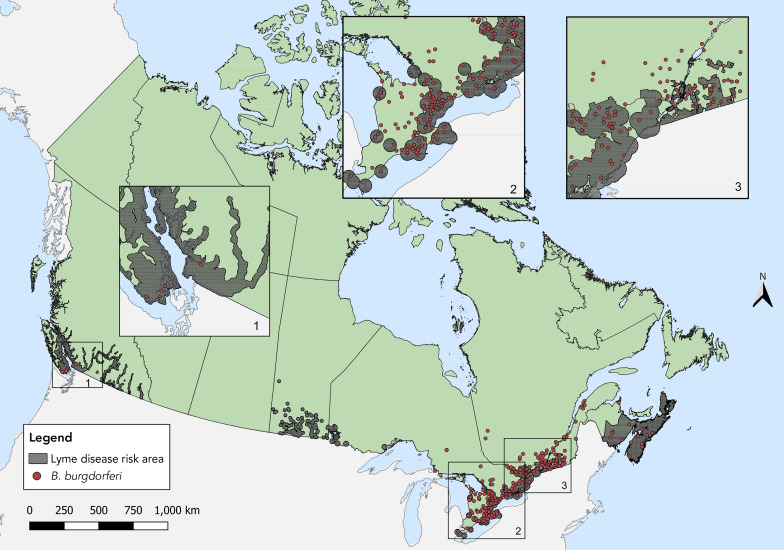
*Ixodes scapularis* and *Ixodes pacificus* ticks submitted through passive surveillance infected with *Borrelia burgdorferi*, Canada, 2021^a,b^ ^a^ Each dot represents the probable location of acquisition of at least one *I. scapularis* (n=510) or *I. pacificus* (n=5) submitted through passive surveillance that was infected with *B. burgdorferi*. The inlays zoom in on regions in British Columbia (Inlay 1) and parts of Ontario (Inlay 2) and Ontario and Québec (Inlay 3) where these ticks were found ^b^ Lyme disease risk areas are identified by the provinces as of 2021 using the methods described in the 2016 national Lyme disease case definition ((20)). On the map, risk areas are identified as hatched grey areas

**Figure 4:**
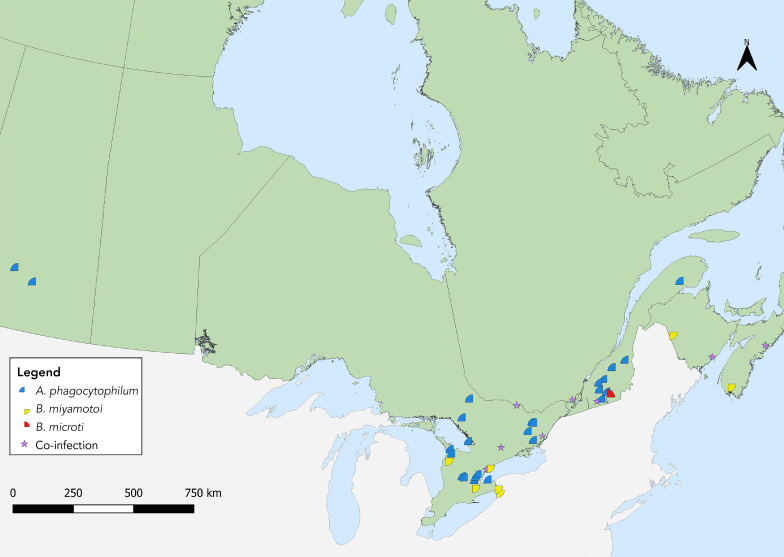
*Ixodes scapularis* ticks with associated pathogens (*Anaplasma phagocytophilum*, *Borrelia miyamotoi*, *Babesia microti*) and co-infections collected through passive surveillance, Canada, 2021^a^ ^a^ Each symbol represents the probable location of acquisition of an *I. scapularis* single or multiple tick submission submitted through passive surveillance that tested positive for *A. phagocytophilum* (n=33), *B. microti* (n=1), *B. miyamotoi* (n=11) or a coinfection. Coinfections were limited to only single submissions of ticks and include *A. phagocytophilum* + *B. burgdorferi* (n=7), *B. burgdorferi* + *B. miyamotoi* (n=2) and one triple coinfection including all three pathogens, all in *I. scapularis*

Ticks infected with tick-borne pathogens were primarily found in Southern and Eastern Ontario, Southern Québec, New Brunswick and in Nova Scotia (**Figure 3**, **Figure 4**). Of the seven provinces where sample-based submissions were included in our analysis, *B. burgdorferi*-infected tick specimens were found in five: British Columbia, Ontario, Québec, New Brunswick and Nova Scotia (Table 4).

*Anaplasma phagocytophilum* was found in *I. scapularis* in all provinces where ticks were tested except Alberta; *B. burgdorferi* in all except Alberta and Saskatchewan (infection prevalence of 1.1% and 18.6%, respectively) (Figure 3, Figure 4, Table 4). *Borrelia miyamotoi* was found in Ontario, Québec, New Brunswick and Nova Scotia, while a single *Babesia microti*-infected tick was found in Québec.

### Active surveillance tick characteristics

In 2021, *I. scapularis* (n=1,935) were collected and tested from five provinces: New Brunswick (n=475), Ontario (n=850), Québec (n=393), Manitoba (n=119) and Nova Scotia (n=98). Of these, the majority of specimens were adults and nymphs followed by larvae (14; 0.7%). In addition, 18 *I. pacificus* were collected in British Columbia.

### Infection prevalence in active surveillance

Laboratory testing results for at least one pathogen were available for 99.5% of *I. scapularis*. The most prevalent pathogen was *B. burgdorferi*, present in all five provinces where *I. scapularis* were collected through active surveillance: Manitoba, Ontario, Québec, New Brunswick and Nova Scotia (**Table 5**). *Borrelia burgdorferi* was detected in 22.3% of ticks tested, compared to 29.3% in 2020 ((6)).

**Table 5:** Prevalence of *Anaplasma phagocytophilum*, *Babesia microti*, *Babesia odocoilei*, *Borrelia burgdorferi*, *Borrelia miyamotoi* and Powassan virus infection in Ixodes scapularis ticks submitted through active surveillance, by province, Canada, 2021^a,b^

Province	Infection prevalence												
	*A. phagocytophilum*		*B. microti*		*B. odocoilei*		*B. burgdorferi*		*B. miyamotoi*		Powassan virus	
	Proportion of positive ticks^c^	%	Proportion of positive ticks^c^	%	Proportion of positive ticks^c^	%	Proportion of positive ticks^c^	%	Proportion of positive ticks^c^	%	Proportion of positive ticks^c^	%
Manitoba	6/119	5.04	1/119	0.84	6/119	5.04	36/119	30.25	1/119	0	1/119	0.84
Ontario	36/834	4.32	0/641	0.00	60/641	9.36	247/843	29.30	4/648	0.62	0/641	0.00
Québec	7/391	1.79	0/391	0.00	57/391	14.58	62/391	15.86	2/391	0.51	0/391	0.00
New Brunswick	29/475	6.11	1/475	0.21	64/475	13.47	68/475	14.32	5/475	1.05	1/475	0.21
Nova Scotia	5/98	5.10	0/98	0.00	16/98	16.33	17/98	17.35	0/98	0	0/98	0.00
Total	83/1917	4.33	2/1724	0.12	203/1724	11.7	430/1926	22.33	12/1733	0.69	2/1724	0.12

^a^ Only results from tests on *I. scapularis* are included. No *I. scapularis* ticks were collected or tested from sites in Alberta, Saskatchewan, Newfoundland and Labrador, Prince Edward Island, Yukon, Northwest Territories or Nunavut

^b^ Infection prevalence is influenced by varying numbers of sites for active surveillance between provinces and seasonal variation when active surveillance took place. Infection prevalence should be interpreted with caution as not all active surveillance conducted in 2021 in Canada were included in this table

^c^ The proportion of positive ticks represents the number of ticks tested positive for the specified pathogen over the total number of ticks tested

**Figure 5:**
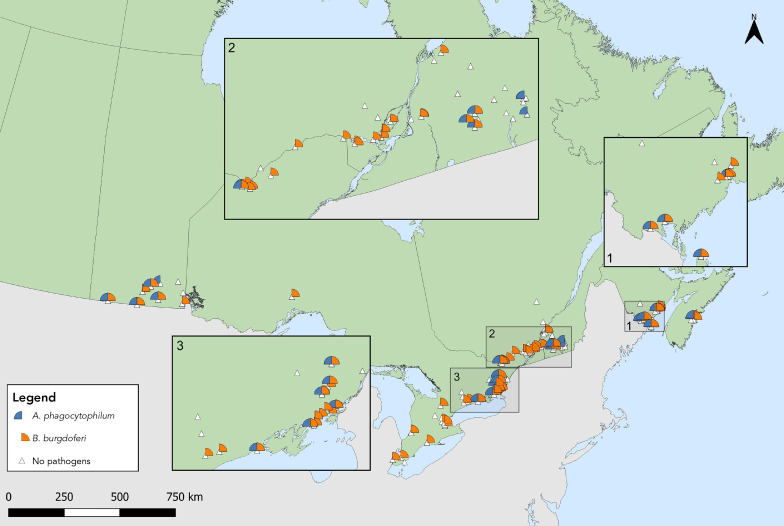
*Ixodes scapularis* ticks with associated pathogens (*Anaplasma phagocytophilum* and *Borrelia burgdorferi*) collected through active surveillance, Canada, 2021^a,b^ ^a^ Each symbol represents an active surveillance site where *A. phagocytophilum* (n=83) or *B. burgdorferi* (n=430) were found in *I. scapularis* ticks. Sites were mapped based on best available information and do not represent precise locations of tick acquisition. The inlays zoom in on regions in New Brunswick (Inlay 1), Québec and Ontario (Inlay 2) and Ontario (Inlay 3) where these ticks were found close together ^b^ No pathogens were found among 18 *I. pacificus* ticks tested from six sites in British Columbia. Map has been zoomed-in for better visibility of tick-borne pathogen distribution

**Figure 6:**
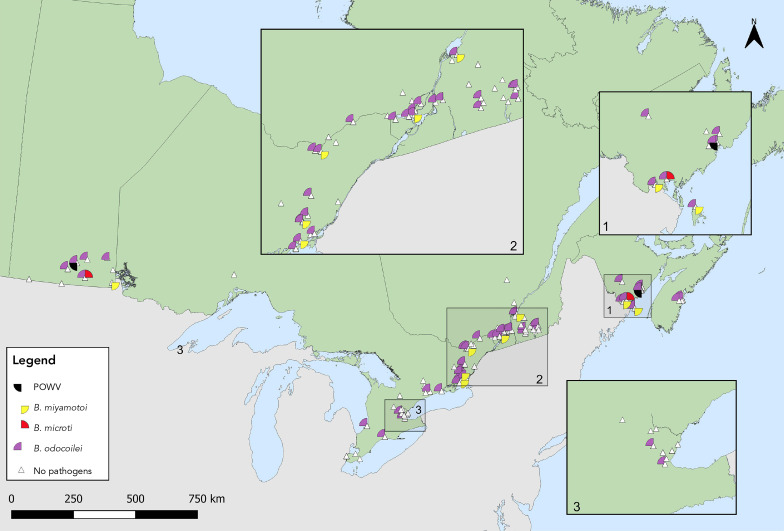
*Ixodes scapularis* ticks with associated pathogens (Powassan virus, *Borrelia miyamotoi*, *Babesia microti* and *Babesia odocoilei*) collected through active surveillance, Canada, 2021^a,b^ Abbreviation: POWV, Powassan virus ^a^ Each symbol represents an active surveillance site where POWV (n=2), *B. miyamotoi* (n=12), *B. microti* (n=2) or *B. odocoilei* (n=203) were found in *I. scapularis* ticks. Sites were mapped based on best available information and do not represent precise locations of tick acquisition. The inlays zoom in on regions in New Brunswick (Inlay 1), Québec and Ontario (Inlay 2) and Ontario (Inlay 3) where these ticks were found close together ^b^ No pathogen was found among 18 *I. pacificus* ticks tested from six sites in British Columbia. Map has been zoomed-in for better visibility of tick-borne pathogen distribution

*Babesia odocoilei*- and *A. phagocytophilum*-infected *I. scapularis* ticks were found in the same five provinces, with overall prevalences of 11.8% and 4.3%, respectively (Table 5, **Figure 5**, **Figure 6**). The overall infection prevalence of the remaining pathogens was less than 1.0% in *I. scapularis*: Twelve *B. miyamotoi*-positive ticks were collected from Manitoba (n=1), Ontario (n=4), Québec (n=2) and New Brunswick (n=5) (Table 5, Figure 6). *Babesia microti*-positive ticks (n=2) and Powassan virus positive ticks (n=2) were found in Manitoba and New Brunswick (Table 5, Figure 6). Among 18 *I. pacificus* ticks collected from six sites in British Columbia, no pathogens were detected.

## Discussion

This report provides an update on the national epidemiology of *I. scapularis* and *I. pacificus* ticks, previously published in 2019 and in 2020 ((6,16)). In 2021, there were 6,892 *I. scapularis* and 777 *I. pacificus* submitted in passive surveillance from ten provinces.

In active surveillance, 1,929 *I. scapularis* and 18 *I. pacificus* were collected in six provinces: British Columbia; Manitoba; Ontario; Québec; New Brunswick; and Nova Scotia. Testing identified the presence of *A. phagocytophilum*, *B. burgdorferi*, *B. miyamotoi*, *B. microti*, *B. odocoilei* and Powassan virus in *I. scapularis*.

Through passive surveillance, 3,459 ticks were sample-based submissions, 41% fewer than the 5,899 ticks submitted as samples in 2020 ((6)). This is likely a result of the discontinuation of passive surveillance programs. As noted in the 2020 tick surveillance report, this could also be due to the continuing effects of COVID-19 pandemic restrictions on traditional passive surveillance, as health units, medical and veterinary clinics could accept fewer physical tick specimens. During 2021, 54% of all passive surveillance data were from eTick, compared to 29% in the previous year ((6)). Active surveillance was also affected by pandemic restrictions, as in-person activities such as field surveillance were limited in Prince Edward Island and Newfoundland and Labrador.

Ticks submitted through passive surveillance followed distinct species-specific temporal patterns ((6)). The bimodal peaks for *I. scapularis* adults observed between May and November were consistent with those seen historically in Central and Eastern Canada ((21–23)) and for *I. pacificus* as observed in the past in British Columbia ((16)) and the Western United States ((24)).

However, tick stage development appears to be occurring earlier in the tick season than observed in previous years. There also seems to be a prolongation of the tick season, with individuals reporting tick exposure throughout the year in passive surveillance data. Thus, the overall risk of tick-borne diseases is increasing due to the temporal and spatial expansion of tick activity, though this will also depend on tick infection prevalence in a given area and individual use of preventative measures. These trends should be monitored in the coming years to determine if the shift in tick submissions is due to weather or other factors, or if it reflects selection bias from current surveillance methods.

The proportion of ticks submitted from dogs or cats continued to increase, almost doubling from 15.1% in 2020 to 26.0% in 2021 ((6)). Like 2020, this is in part due to the inclusion of data from eTick, which does not have any host-based restrictions. The inclusion of eTick data may also have contributed to a shift in the distribution of the life stage of ticks collected; for example, nearly three times the number of nymphs were collected in passive surveillance during 2021 compared with 2020 ((6)).

The national estimates for prevalence for each pathogen through passive surveillance, except *B. miyamotoi* in *I. scapularis*, were slightly higher than the results for 2020 ((6)). *Borrelia burgdorferi* was detected in 18.6% of *I. scapularis* compared to 17.2% in 2020, *A. phagocytophilum* in 1.1% compared to 0.9%, *B. microti* in 0.04% compared to 0.02% and *B. miyamotoi* in 0.4% compared to 0.5% in 2020 ((6)). Among *I. pacificus* ticks, 0.9% were positive for *B. burgdorferi* compared to none in 2020.

For active surveillance, infection prevalence results were similar to those obtained in 2020 for all pathogens except *B. burgdorferi*, which was less prevalent in 2021 in Ontario, Québec and New Brunswick ((6)). This may be partially explained by the larger total tick numbers collected during 2021; 3.5 times for Québec and 6.5 times for Ontario in 2021 versus 2020 ((6)). Other factors that influence infection prevalence estimates from year-to-year or between provinces include variation in sites selected and their ecological and host-related characteristics ((25)).

Our results also include the infection prevalence of *B. odocoilei*, indicating a prevalence close to 15% in samples tested from each of Québec, New Brunswick and Nova Scotia. It was also present in other provinces where it was tested for, namely, Manitoba and Ontario.

## Strengths and limitations

While several traditional passive surveillance programs have been phased out, incorporating data from eTick allows us to continue monitoring the geographic presence of these vectors across the country. Combining passive and active surveillance information allows the strengths and weaknesses of the systems to complement each other. While active surveillance is limited in geographic and temporal scope, passive surveillance programs are not limited to specific site locations so data can be gathered from large areas throughout the year.

As noted in 2020, COVID-19 pandemic restrictions affected public health surveillance efforts in 2021. Second, tick specimens collected from eTick, though useful for consistent geographic surveillance, are not routinely requested for tick-borne pathogen testing ((15)). Recall bias in reporting travel history and other variables in passive surveillance might create uncertainty. For active surveillance, it is likely that other programs were conducted in 2021 that did not submit ticks for pathogen testing at NML and were not included in this summary.

## Conclusion

Despite data limitations and resource constraints, efforts in tick surveillance over time have permitted the identification of increasing prevalence and emergence of tick-borne disease pathogens in Canada. Healthcare professionals and the public should be reminded that there is a risk of exposure to infected ticks outside of Lyme disease at-risk areas, even if the risk is low in those areas. Tick surveillance data are an important source of information for public health authorities as they endeavour to identify risk areas, target prevention and education efforts.
